# Supplementary material for: Pulmonary fibroblasts activated by the addition of TNF-α and IL-4 enhance lymphangiogenic capacity and ameliorate lung fibrosis in an allogeneic rat model
Source: PLoS One. 2026 Feb 10;21(2):e0342528. doi: 10.1371/journal.pone.0342528 (PMC12890169; doi:10.1371/journal.pone.0342528)
Supplement: S1 Table — (PDF) [file pone.0342528.s001.pdf]

S1 Table. Antibodies and reagents used for FACS and immunohistochemistry.

Product	Supplier	Reference	Application
CD106 (VCAM-1)-Biotin, human	Miltenyi Biotec	130-104-123	FACS
CD90-PE, human	Miltenyi Biotec	130-114-860	FACS
BV421 mouse Ig1 Isotype control	BD Biosciences	562438	FACS
REA control (S)-PE	Miltenyi Biotec	130-113-438	FACS
Anti-VE Cadherin	abcam	ab33168	immunohistochemistry
Anti-Vimentin	abcam	ab20346	immunohistochemistry
Goat Anti- Rabbit IgG H&L (Alexa Fluor 488)	abcam	ab150077	immunohistochemistry
Goat Anti- Mouse IgG H&L (Alexa Fluor 647)	abcam	ab150115	immunohistochemistry
Anti-HLA DR + DP + DQ	abcam	ab23901	FACS
CD80-PE, human	Miltenyi Biotec	130-123-324	FACS
CD86-PE, human,	Miltenyi Biotec	130-116-263	FACS
Human HLA Class I APC-conjugated	R&D Systems	FAB7098A	FACS
Mouse IgG2A APC-conjugated	R&D Systems	IC003A	FACS
PE Mouse IgG2a [X5563] - Isotype Control	abcam	ab91363	FACS
REA control (S)-PE, clone: REA293	Miltenyi Biotec	130-113-438	FACS
Mouse Anti-Rat CD106	BD Biosciences	559165	FACS
APC Goat anti-mouse IgG	BioLegend	405308	FACS
Goat Anti-Wheat Germ Agglutinin(WGA)	Vector	AS-2024-1	

Donkey Anti-Goat IgG H&L	abcam	Ab150129	
