# Supplementary material for: Pulmonary fibroblasts activated by the addition of TNF-α and IL-4 enhance lymphangiogenic capacity and ameliorate lung fibrosis in an allogeneic rat model
Source: PLoS One. 2026 Feb 10;21(2):e0342528. doi: 10.1371/journal.pone.0342528 (PMC12890169; doi:10.1371/journal.pone.0342528)
Supplement: S2 Table — (PDF) [file pone.0342528.s002.pdf]

S2 Table. Primers and Probes used for RT-qPCR

Gene	Forward Primer	Reverse Primer
**ADM**	ATGTACCTGGGTTCGCTCGC	CCACGACTCAGAGCCCACTT
**VEGF-C**	GCTACCTCAGCAAGACGTTATTTG	ATCGGCAGGAAGTGTGATTG
**β-Actin**	ACCTTCTACAATGAGCTGCG	CCTGGATAGCAACGTACATGG
**TNF-α**	GCGCTCCCCAAAAAGATGGG	GGACCGATCACCCCGAAGTTC
**CCL2(MCP-1)**	CGCTTCTGGGCCTGTTGTTC	CTCCAGCCGACTCATTGGGA
**IL-6**	GCCAGTTGCCTTCTTGGGAC	AGTAGGGAAGGCAGTGGCTG
**TGF-β**	CCCCTGGAAAGGGCTCAACA	GCTGCCGTACACAGCAGTTC
**Acta2(α-SMA)**	GAGGAGCATCCGACCTTGCTA	GAGGGACAGCACAGCCTGAA
**Col1a1**	TCAGCCCAAACCCCAAGGAG	TAGCGACATCGGCAGGATCG
**Lyve-1**	CGGTTCTCAGTCATCCCTCGG	GTTGGCCCAGGTGTCAGATG
**Flt4(VEGFR3)**	AGATGCGATGTCCGGTAGCC	CCAGGTCGATTCCCGACTCT
