# Supplementary material for: Pulmonary fibroblasts activated by the addition of TNF-α and IL-4 enhance lymphangiogenic capacity and ameliorate lung fibrosis in an allogeneic rat model
Source: PLoS One. 2026 Feb 10;21(2):e0342528. doi: 10.1371/journal.pone.0342528 (PMC12890169; doi:10.1371/journal.pone.0342528)
Supplement: Attachment — Submitted filename: Peer review-PLOS ONE.docx [file pone.0342528.s003.docx]

**Dear Editor,**

Thank you for your message. I appreciate the opportunity to contribute to the review process.

Here are my comments on the manuscript **PONE-D-25-47407**, entitled *“Pulmonary fibroblasts activated by the addition of TNF-α and IL-4 enhance lymphangiogenic capacity and ameliorate lung fibrosis in an allogeneic rat model.”*

The study is interesting and explores a novel approach to reduce lung fibrosis using cytokine-stimulated pulmonary fibroblasts. The experiments were appropriately conducted, but some points need clarification or improvement before publication.

**Major Comments**

1. **Mechanistic Evidence of Lymphangiogenesis**
   - LYVE-1 and VEGFR3 upregulation is shown, but it is unclear whether these structures are true lymphatic vessels or newly formed versus recruited from endogenous cells.
   - **Suggestion:** Clarify to confirm lymphangiogenesis, or discuss this consideration in the **Limitations** section.
2. **References**
   - References in the text are not formatted according to the journal’s guidelines.
   - **Suggestion:** Please adjust all in-text citation numbers and the reference list to comply with **PLOS ONE**formatting requirements.
3. **SP-D and Fibrinogen**
   - These markers also decrease in saline controls.
   - **Suggestion:** Discuss natural recovery versus treatment effect.

**Overall Recommendation:** Minor Revision
