# Supplementary material for: Pulmonary fibroblasts activated by the addition of TNF-α and IL-4 enhance lymphangiogenic capacity and ameliorate lung fibrosis in an allogeneic rat model
Source: PLoS One. 2026 Feb 10;21(2):e0342528. doi: 10.1371/journal.pone.0342528 (PMC12890169; doi:10.1371/journal.pone.0342528)
Supplement: Attachment — Submitted filename: Response to Reviewers.docx [file pone.0342528.s005.docx]

Response to Reviewers

Manuscript ID: PONE-D-25-47407

**Title: Pulmonary fibroblasts activated by the addition of TNF-α and IL-4 enhance lymphangiogenic capacity and ameliorate lung fibrosis in an allogeneic rat model**

We sincerely thank the Academic Editor and the reviewers for their careful evaluation of our manuscript and for their constructive and insightful comments. We have carefully considered all comments and have revised the manuscript accordingly to improve clarity, balance of interpretation, and compliance with PLOS ONE editorial requirements.

For ease of review, our responses are organized as follows:

•	Responses to Journal Requirements

•	Responses to Additional Editor Comments

•	Responses to Reviewer #1

•	Responses to Reviewer #2

All newly added or revised text in the manuscript has been highlighted in yellow for ease of reference. Minor editorial changes and clarifications have been highlighted in green.

# Response to the Academic Editor

Comment 1: Immunogenicity and Allogeneic Use — Need for Further Characterization

The manuscript reports “minimal immunogenicity” of cytokine-stimulated PFs based on the absence of HLA-II and co-stimulatory molecules (CD80/86). While this aligns with characteristics observed in MSC-based therapies, such in vitro findings do not fully predict in vivo immune responses. Allogeneic cell transplantation can still lead to immunological events such as allo-antibody formation, innate immune activation, or delayed rejection. A more comprehensive discussion of these potential risks—along with considerations regarding long-term engraftment, persistence, and possible tumorigenicity—would substantially strengthen the manuscript.

Furthermore, clarification is needed regarding the long-term stability and fate of transplanted PFs. It remains unclear whether cytokine-stimulated PFs sustain a lymphangiogenic phenotype over time or undergo phenotypic drift, dedifferentiation, or aberrant contributions to tissue remodeling after transplantation.

Response:

Thank you for this important comment. We agree that in vitro immunogenicity data do not fully predict in vivo responses, and that discussion of long-term safety is essential.

We have added two new paragraphs to the Discussion (Page 17, Line 8-Page 18, Line 2) to address these concerns:

- Immunogenicity and safety: We now explicitly acknowledge that allogeneic cell transplantation may elicit immune responses, including allo-antibody formation, innate immune activation, and delayed rejection, even when in vitro immunophenotyping suggests low immunogenicity. We also discuss tumorigenicity and note that no evidence suggestive of malignant transformation was observed in the present short-term culture and observation period; however, we emphasize that dedicated long-term tumorigenicity and immunogenicity studies will be required in accordance with regulatory guidelines. In addition, we discuss the possibility that the observed therapeutic effects are mediated predominantly through early paracrine signaling prior to immune-mediated clearance rather than long-term engraftment.
- Phenotypic stability and study limitations: We address the potential for phenotypic drift, dedifferentiation, or environment-driven reprogramming of transplanted PFs within fibrotic tissues. While our gene expression analyses (decreased *COL1A1* and *αSMA*, increased *Lyve1* and *Vegfr3*) are consistent with a lymphangiogenic-associated phenotype during the observation period, we acknowledge that these data alone cannot definitively establish long-term phenotypic stability. We therefore clearly state the limitations of our 2-week observation period and the absence of comprehensive immunological monitoring or lineage-tracing analyses, and propose future studies to directly address cell fate, persistence, and long-term safety.

We believe these revisions address the reviewer's concerns while being honest about the study's limitations.

Comment 2: Specificity of the Observed Effects — Role of PFs vs. Other Cell Populations

The observed increases in lymphatic marker expression (LYVE-1, VEGFR3) and the reduction in fibrosis are attributed to the transplanted PFs. However, existing literature indicates that lymphangiogenesis during fibrosis often involves endogenous mechanisms, including activation of resident lymphatic endothelial cells or macrophage-derived lymphatic-like cells. Alternative explanations—such as host-mediated lymphatic remodeling or macrophage transdifferentiation—should be acknowledged and discussed.

If feasible, lineage tracing or similar techniques would provide stronger evidence regarding whether transplanted PFs directly differentiate into lymphatic endothelial-like cells or contribute structurally to lymphatic remodeling, rather than acting solely through paracrine signaling.

Response:

We thank the reviewer for this insightful comment. We agree that the observed increases in lymphatic marker expression and the reduction in fibrosis cannot be attributed solely to direct differentiation or structural incorporation of transplanted PFs without considering host-mediated mechanisms.

We have added a new paragraph to the Discussion (Page 18, Line 8-Page 18, Line 25) to explicitly address these alternative explanations. In this section, we acknowledge that lymphangiogenesis during fibrosis can involve activation and expansion of resident lymphatic endothelial cells, as well as contributions from macrophage-derived lymphatic-like cells and broader host-driven tissue remodeling processes, as reported in the literature.

We further discuss that, in the absence of lineage tracing or cell fate–mapping approaches, it is not possible to definitively distinguish whether transplanted PFs directly acquire lymphatic endothelial–like characteristics or whether the observed lymphangiogenic responses are primarily mediated by host cells. Consistent with our previous work demonstrating that cytokine-stimulated fibroblasts promote lymphangiogenesis predominantly through ADM-mediated paracrine signaling (reference), we propose that paracrine modulation of the host microenvironment represents a plausible and likely mechanism in the present study. However, we clearly state that this interpretation remains inferential, and that future studies incorporating lineage tracing or similar approaches will be required to directly assess the cellular origin and structural contribution to lymphatic remodeling.

Comment 3: Quantitative Strength of Findings and Statistical Interpretation

Several results are described as demonstrating a “greater inhibitory trend,” particularly in the rPF+CKs group compared to rPF alone; however, these differences were not statistically significant. Such findings should be presented explicitly as trends and not interpreted as definitive effects. Similarly, although increases in lymphatic markers and ISH signals are reported, the manuscript acknowledges that the increase in LYVE-1 ISH did not reach statistical significance. These limitations should be more transparently addressed, ideally in both the Results and Discussion sections.

Interpretation of plasma biomarker trends (SP-D, fibrinogen) should also be tempered by the known variability of the bleomycin model and its partial spontaneous resolution. Overstating these findings as clear therapeutic effects may be misleading without stronger statistical support.

Response:

We thank the reviewer for this important comment regarding statistical rigor and interpretation of the data. We agree that non-significant differences should be presented explicitly as trends and not interpreted as definitive effects.

We have revised the manuscript accordingly in both the Results and Discussion sections.

**Results section revisions:**

- Page 13, Line 19-21: We revised the description of fibrotic markers (*IL6, αSMA, Col1a1*) to explicitly state that although reductions were observed in both treatment groups, the differences between rPF and rPF+CKs did not reach statistical significance and should be interpreted as trends. Language implying a definitive or superior inhibitory effect of rPF+CKs was removed.
- Page 13, Line 22-24: For lymphatic markers, we clarified that while *Vegfr3* showed a statistically significant increase in the rPF+CKs group, the increase in *Lyve1* ISH signal did not reach statistical significance and is therefore described as a trend rather than a definitive effect.

**Discussion section revisions:**

- Page 19, Line 8-14: We added a dedicated limitations paragraph following the histological results to explicitly acknowledge that several outcomes, including fibrotic markers and *Lyve1* ISH signals, demonstrated non-significant trends rather than statistically robust effects. We discuss the limited statistical power related to sample size, the short 2-week observation period, and the inherent variability and partial spontaneous resolution characteristic of the bleomycin model.
- Page 19, Line 14-20: Interpretation of plasma biomarkers (SP-D and fibrinogen) was further tempered to emphasize that observed trends should be interpreted cautiously and not as definitive therapeutic effects, given the known variability of these markers in the bleomycin-induced lung injury model.

These revisions ensure transparent presentation of statistically significant findings while appropriately contextualizing non-significant trends and model-related limitations.

# Response to Reviewer #1

Comment:

Excellent manuscript regarding cytokine-stimulated pulmonary fibroblasts. Pulmonary fibroblasts may serve as a novel cell source for antifibrotic therapy by modulating lymphangiogenesis and tissue remodeling, providing a potential alternative to conventional stem cell-based approaches for fibrotic lung diseases. This may be a new molecular pathway for potential treatment targets in pulmonary hypertension.

Response:

We sincerely thank the reviewer for the positive and encouraging evaluation of our manuscript. We greatly appreciate the reviewer’s recognition of the novelty and potential therapeutic significance of cytokine-stimulated pulmonary fibroblasts as an antifibrotic strategy. These supportive comments were very helpful in strengthening our confidence in the conceptual framework and presentation of this study.

# Response to Reviewer #2

General Comment:

I would like to thank you and your colleagues for this excellent scientific work and for your valuable contribution to the advancement of science.

Response:

We thank the reviewer for the positive assessment of our work and for the encouraging comments. We appreciate the reviewer’s thoughtful evaluation and support of our study.

Major Comment 1: Mechanistic Evidence of Lymphangiogenesis

- LYVE-1 and VEGFR3 upregulation is shown, but it is unclear whether these structures are true lymphatic vessels or newly formed versus recruited from endogenous cells.
- Suggestion: Clarify to confirm lymphangiogenesis, or discuss this consideration in the Limitations section.

Response:

We thank the reviewer for this important comment regarding whether the observed *Lyve1*– and *Vegfr3*-positive structures represent true lymphatic vessels and whether they arise from newly formed lymphangiogenesis or recruitment of endogenous cells.

We have addressed this consideration in the Discussion (Page 18, Line 8-25) by explicitly acknowledging multiple potential mechanisms underlying the observed LYVE-1 and VEGFR3 upregulation, including activation and expansion of resident lymphatic endothelial cells, macrophage-derived lymphatic-like cells, and host-driven tissue remodeling processes. We further clarify that, in the absence of lineage tracing or structural validation approaches, we cannot definitively determine whether the LYVE-1– and VEGFR3-positive structures represent newly formed lymphatic vessels or reflect recruitment and remodeling of endogenous lymphatic networks.

Accordingly, we have discussed this uncertainty as a limitation of the current study. Consistent with our previous work demonstrating predominantly paracrine mechanisms in cytokine-stimulated fibroblasts[1], and considering the transient survival of allogeneic cells, we propose that paracrine activation of host lymphangiogenic pathways is a likely mechanism, while clearly stating that this interpretation remains inferential.

Major Comment 2: References

- References in the text are not formatted according to the journal’s guidelines.
- Suggestion: Please adjust all in-text citation numbers and the reference list to comply with PLOS ONEformatting requirements.

Response:

We thank the reviewer for pointing out the reference formatting issue. We have carefully reviewed and corrected all in-text citation numbers and the reference list to comply with PLOS ONE formatting requirements. All references now follow the journal's guidelines for numbering, punctuation, and bibliographic format.

Major Comment 3: SP-D and Fibrinogen

- These markers also decrease in saline controls.
- Suggestion: Discuss natural recovery versus treatment effect.

Response:

We thank the reviewer for this important observation. We agree that decreases in fibrotic and inflammatory markers observed in saline-treated controls likely reflect the known tendency toward partial spontaneous recovery in the bleomycin-induced lung injury model.

We have addressed this consideration in the Discussion (Page 19, Line 8–21) by explicitly discussing the contribution of natural recovery versus treatment-related effects. In this section, we acknowledge that reductions in markers such as SP-D and fibrinogen were also observed in control animals and therefore cannot be attributed solely to therapeutic intervention. We further clarify that the observed changes in treated groups should be interpreted as trends that may reflect modulation of the recovery process rather than definitive evidence of treatment-driven reversal of fibrosis.

Accordingly, we have revised the Discussion to avoid overinterpretation of these findings and to emphasize the limitations imposed by model variability, partial spontaneous resolution, and the relatively short observation period.

# Response to Journal Requirements

Journal Requirement 1: PLOS ONE formatting and file naming

Please ensure that your manuscript meets PLOS ONE’s style requirements, including those for file naming.

Response:

**Style and File Naming:**

Thank you for this reminder. We have carefully reviewed and revised the manuscript to ensure full compliance with PLOS ONE's style requirements and submission guidelines.

**Reference formatting:**

All in-text citations will be formatted to the bracketed format required by PLOS ONE (e.g., [1], [2-5], [3,7,9]) using our reference management software prior to final submission. References are listed after the main text in numerical order, and references with more than six authors use "et al." format as specified in the journal guidelines.

**Figure and table citations:**

We have revised all figure citations throughout the manuscript to conform to PLOS ONE style (e.g., "Figure. 1A" has been changed to "Fig 1A", "Figure 2" to "Fig 2", etc.). Tables are cited as "Table 1", "Table 2", etc. Figure captions appear directly after the paragraphs in which they are first cited, with bold type used for figure titles.

**Heading format:**

All section headings use the appropriate PLOS ONE formatting:

- Level 1 headings (Abstract, Introduction, Materials and methods, Results, Discussion, etc.): Bold, 18pt font, sentence case
- Level 2 and Level 3 headings: Appropriately formatted with bold type and sentence case

**File naming:**

All files will be named according to PLOS ONE conventions upon submission (Fig1.tif, Fig2.tif, S1_Fig.tif, etc.).

**Manuscript formatting:**

The manuscript body is formatted in double-space paragraph format as required by PLOS ONE submission guidelines.

We believe the manuscript now fully conforms to all PLOS ONE style and formatting requirements.

Journal Requirement 2: Animal ethics and humane endpoints

Please report additional details in the Methods section regarding animal care and use, including methods of sacrifice, anesthesia/analgesia, and efforts to alleviate suffering. Please also complete and upload the Humane Endpoints Checklist.

Response:

**Journal Requirement 2: Animal Ethics and Humane Endpoints**
Thank you for this requirement. We have extensively revised and expanded the Animal experiments section in Materials and Methods (Page 4, Line 19-Page 6, Line 5, now titled "Animal welfare and experimental procedures").

**The revised section now includes**

- Complete details of animal housing conditions (temperature, humidity, light cycle, acclimatization period)
- Full anesthesia protocol including the triple-combination anesthetic cocktail composition, dose (5 mL/kg), and route of administration; bleomycin dose (5 mg/kg) by intratracheal micro-spray; and justification for not using additional post-operative analgesia
- Prospective humane endpoint criteria (body weight loss >20%, severe respiratory distress, inability to access food/water, severe lethargy), daily monitoring protocols, and confirmation that no animals reached these endpoints
- Detailed euthanasia method (exsanguination under deep isoflurane anesthesia) with confirmation of death, in accordance with AVMA guidelines
- Explicit discussion of efforts to minimize suffering, including justification for bleomycin dose and observation period selection

We have also completed and uploaded the Humane Endpoints Checklist as requested.

Journal Requirement 3: Funding Statement and Competing Interests

Please provide an amended Funding Statement and updated Competing Interests Statement reflecting the commercial affiliation with LYMPHOGENiX Ltd, including clarification of the role of funders and confirmation of adherence to PLOS ONE data and materials sharing policies.

Response:

Thank you for this requirement. We have revised both the Funding Statement and Competing Interests Statement to accurately reflect the commercial affiliation with LYMPHOGENiX Ltd.

**Revised Funding Statement:**

This research was supported through a collaborative research agreement with LYMPHOGENiX Ltd. LYMPHOGENiX Ltd. provided support in the form of salaries for authors YM and TI, and covered research-related expenses including personnel costs. All aspects of the study, including study design, experimental execution, data collection, data analysis, and interpretation of results, were conducted independently at Nagoya University, where the study was performed. LYMPHOGENiX Ltd. did not have any additional role in the study design, data collection and analysis, decision to publish, or preparation of the manuscript. The specific roles of these authors are articulated in the 'author contributions' section.

**Revised Competing Interests Statement:**

YM and TI are employed by/affiliated with LYMPHOGENiX Ltd. This does not alter our adherence to PLOS ONE policies on sharing data and materials.

These revisions clarify that LYMPHOGENiX Ltd. provided financial support through a collaborative research agreement, including salaries for authors YM and TI, while the research was conducted independently at Nagoya University. We confirm full adherence to PLOS ONE data and materials sharing policies.

Journal Requirement 4: Citation of recommended literature

If reviewer comments include recommendations to cite specific previously published works, please evaluate their relevance.

Response:

We have carefully reviewed all reviewer comments. Neither the Academic Editor nor Reviewer 2 recommended citation of specific previously published works. All reviewer comments focused on scientific interpretation, data presentation, and methodological considerations rather than literature citation. Therefore, no additional literature citations were required beyond those we added to address the substantive scientific issues raised in Comments 1 and 2.

Finally, we thank the Academic Editor and the reviewers for their thoughtful and constructive comments.

We believe that the revisions have improved the clarity and balance of the manuscript,

and we hope that the revised version satisfactorily addresses all concerns raised.

**References**

1.	Matsuoka Y, Shimizu Y, Luo H, Segard BD, Matsuyama M, Hayashi T, et al. Adrenomedullin production by adult cardiac fibroblasts via NF-κB/STAT6 signaling enhances post-infarction lymphangiogenesis and cardiac repair. Sci Rep. 2025;15. doi:10.1038/s41598-025-17224-6
